# Aptamers as Novel Binding Molecules on an Antimicrobial Peptide-Armored Composite Hydrogel Wound Dressing for Specific Removal and Efficient Eradication of *Pseudomonas aeruginosa*

**DOI:** 10.3390/ijms24054800

**Published:** 2023-03-02

**Authors:** Markus Kraemer, Magali Bellion, Ann-Kathrin Kissmann, Tilmann Herberger, Christopher V. Synatschke, Anil Bozdogan, Jakob Andersson, Armando Rodriguez, Ludger Ständker, Sebastien Wiese, Steffen Stenger, Barbara Spellerberg, Kay-Eberhard Gottschalk, Ahmet Cetinkaya, Joanna Pietrasik, Tanja Weil, Frank Rosenau

**Affiliations:** 1 Institute of Pharmaceutical Biotechnology, Ulm University, Albert-Einstein-Allee 11, 89081 Ulm, Germany; 2 Max-Planck-Institute for Polymer Research Mainz, Ackermannweg 10, 55128 Mainz, Germany; 3 Center for Electrochemical Surface Technology (CEST), Austrian Institute of Technology, 3420 Tulln, Austria; 4 Austrian Institute of Technology, Giefinggasse 4, 1210 Vienna, Austria; 5 Core Facility for Functional Peptidomics, Ulm Peptide Pharmaceuticals (U-PEP), Faculty of Medicine, Ulm University, 89081 Ulm, Germany; 6 Core Unit of Mass Spectrometry and Proteomics, Faculty of Medicine, Ulm University, 89081 Ulm, Germany; 7 Institute for Medical Microbiology and Hygiene, University Hospital Ulm, 89081 Ulm, Germany; 8 Institute of Experimental Physics, Ulm University, Albert-Einstein-Allee 11, 89081 Ulm, Germany; 9 Institute of Polymer and Dye Technology, Lodz University of Technology, Stefanowskiego 16, 90-537 Lodz, Poland

**Keywords:** aptamer, focused library, antimicrobial peptide, next-generation wound dressing

## Abstract

Here we present for the first time a potential wound dressing material implementing aptamers as binding entities to remove pathogenic cells from newly contaminated surfaces of wound matrix-mimicking collagen gels. The model pathogen in this study was the Gram-negative opportunistic bacterium *Pseudomonas aeruginosa*, which represents a considerable health threat in hospital environments as a cause of severe infections of burn or post-surgery wounds. A two-layered hydrogel composite material was constructed based on an established eight-membered focused anti-*P. aeruginosa* polyclonal aptamer library, which was chemically crosslinked to the material surface to form a trapping zone for efficient binding of the pathogen. A drug-loaded zone of the composite released the C14R antimicrobial peptide to deliver it directly to the bound pathogenic cells. We demonstrate that this material combining aptamer-mediated affinity and peptide-dependent pathogen eradication can quantitatively remove bacterial cells from the “wound” surface, and we show that the surface-trapped bacteria are completely killed. The drug delivery function of the composite thus represents an extra safeguarding property and thus probably one of the most important additional advances of a next-generation or smart wound dressing ensuring the complete removal and/or eradication of the pathogen of a freshly infected wound.

## 1. Introduction

In addition to potential global threats such as deadly virus infections, as exemplified by the current SARS-CoV-2 pandemic, infections with pathogenic microorganisms have become one of the most threatening health problems of our time [1,2]. Multi-resistant bacteria or fungi urge mankind to develop novel antimicrobial drugs to compensate for the expiration of currently used antimicrobials, which will otherwise lead to a drastic increase in death cases in the near future [3]. Of special concern are nosocomial, i.e. hospital-acquired, infections that can lead to severe complications in the course of their hospital therapies and can lead to significantly elevated mortality in patients [4]. Immunosuppressed patients are most at risk for these types of infections. According to the World Health Organization (WHO), nosocomial infections are the second leading cause of death in the world and more than four million patients are affected by nosocomial infections every year in Europe alone according to the European Center for Disease Control (ECDC)) [5,6]. These statistics can only be interpreted as a truly alarming wake-up call for medical science. Besides the lung in the case of all types of pneumonia, larger wounds, including burn wounds and those originating as consequences of surgery, represent major gateways for microbes to enter the human body, with an intrinsic risk of developing systemic infections in later stages [7,8]. Consequently, prevention of sepsis (also designated “septicemia”) as a still growing and menacing general problem requires efficient control of infections as early as possible, which may involve the precautionary application of antimicrobial drugs and the meticulous, anti-infective treatment of wounds [9,10]. Wound dressings, in general, may represent the first line of defense against infections of larger lesions and injuries and can play an important role in the immediate management of freshly infected tissue [11,12]. Next-generation wound dressing materials often contain hydrogel constituents responsible for moisture control on the wound surface and additional beneficial functions, including sensors to monitor infections, or equipped with an affinity toward dedicated pathogens allowing their specific immobilization and removal from the wound surface during wound care [13,14,15,16,17].

We have previously introduced composite wound dressing materials based on protein hydrogels functionalized with lectins for the efficient binding of pathogens such as the pathogenic yeast *Candida auris* and the opportunistic Gram-negative bacterium *P. aeruginosa*, which is one of the most important causative agents of severe infections and, as a carbapenem-resistant mutant strain, it is part of an alarming global health threat identified by the WHO [16,17]. Treatment of strains already resistant against last resort antibiotics requires novel antimicrobial drugs, which can be acquired from the heterogenic molecule class of antimicrobial peptides (AMPs) including those from marine animals (e.g., snails) [18,19,20,21,22,23]. These AMPs are expected to require proper delivery in these next-generation wound dressing concepts to successfully combat infections. The 16 amino acid-long antimicrobial peptide C14R (amino acid sequence: CSSGSLWRLIRRFLRR) has proven its antimicrobial activity against carbapenem-resistant *P. aeruginosa* as an antimicrobial drug molecule itself, and also upon release from a two-layered composite of a protein-based affinity hydrogel and a drug carrier compartment fabricated from fibril forming-protected amino acid monomers [17]. Such “capture-and-kill” concepts exemplified with the target pathogen *P. aeruginosa* used lectins as affinity molecules, which can bind specifically to glycosylated cell surface structures of bacteria and higher cells, including yeasts [16,17,24,25]. By contrast, DNA aptamers represent a class of ligand molecules that are evolved in the laboratory directedly by SELEX processes and can have truly remarkable specificities toward dedicated targets [26,27,28]. Polyclonal aptamer libraries, resulting directly from the SELEX process, as well as single aptamers isolated by bioinformatic analyses from these libraries can bind *P. aeruginosa* strains with high specificity, and have been used as binding entities on protein beads for the specific capturing of *P. aeruginosa* from serum and blood [29,30]. The immediate use of such polyclonal libraries originating directly from SELEX processes without the need for subsequent analyses and selection of their individual members is inspired by the concept of using polyclonal antibody preparations instead of monoclonal antibodies. We have previously shown that these libraries can outperform individual aptamers, because multiple epitopes on the target structure are recognized allowing a robust binding of the target regardless of possible alterations in the composition through mutation or the growth phase [29,30].

**Figure 1:**
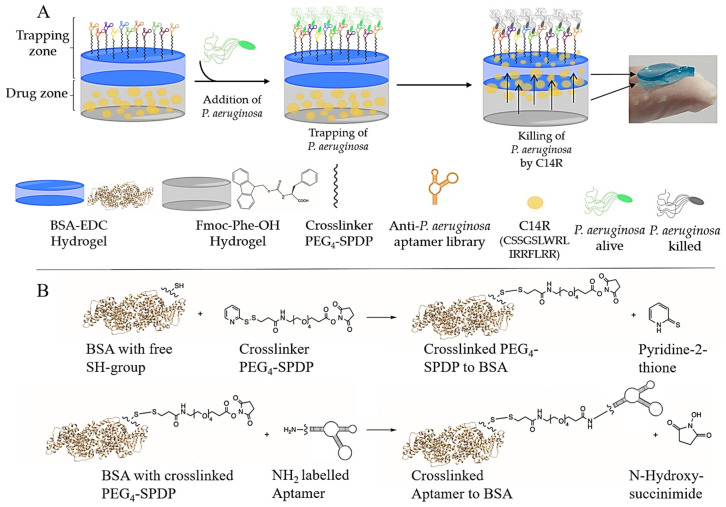
General concept of the *P. aeruginosa*-specific AA-EraGel pathogen killing trap. (**A**) The general concept of the hydrogel trap is in a first step to immobilize the bacteria using the specific aptamer library for *P. aeruginosa* and then to kill the immobilized bacteria with the AMP which is continuously released from the hydrogels. The upper hydrogel consists of bovine serum albumin (BSA) chemically crosslinked with EDC (1-ethyl-3-(3-dimethylaminopropyl)carbodiimide) and then functionalized with a PEG_4_-SPDP (Tetraethylenglycole-Succinimidyl 3-(2-Pyridyldithio)propionatend, an NH_2_-labeled aptamer library specific for *P. aeruginosa*, to irreversibly immobilize the bacteria on the surface of the hydrogel. (**B**) The reaction starts with a displacement reaction of the sulfhydryl-reactive molecule (2-pyridyldithio group) of PEG_4_-SPDP and the free SH group of the BSA–EDC hydrogel. In the next step, the NH_2_-labeled aptamer reacts with the amine-reactive portion (*N*-hydroxysuccinimide) of PEG_4_-SPDP via an NHS ester reaction. The bottom hydrogel consists of Fmoc-phe-OH (*N*-(Fluorenyl-9-methoxycarbonyl)-L-phenylalanin) and phosphate buffer loaded with a specific AMP against *P. aeruginosa*, here C14R.

Here we present a two-layered hydrogel composite material, which makes use of the pool of *P. aeruginosa*-specific aptamers (C1R1, C2R1, C2R2, C2R10, C4R2, C6R3, C10R5 and C10R6) chemically crosslinked to the material surface to form a trapping zone for efficient binding of the pathogen from a minimalized wound model consisting of a collagen-based gel resembling the extracellular matrix in a fresh wound. A drug-loaded zone of the composite released the C14R antimicrobial peptide to deliver it directly to the bound pathogenic cells (Figure 1). We demonstrate that this material combining aptamer-mediated affinity and peptide-dependent pathogen eradication (AA-EraGel) can quantitatively remove bacterial cells from the “wound” surface, and we show that the surface-trapped bacteria are completely killed. The drug delivery function of the composite thus represents an extra safeguarding property and thus probably one of the most important additional advances of a next-generation or smart wound dressing ensuring the complete removal and/or eradication of the pathogen of a freshly infected wound.

## 2. Results

**Figure 2:**
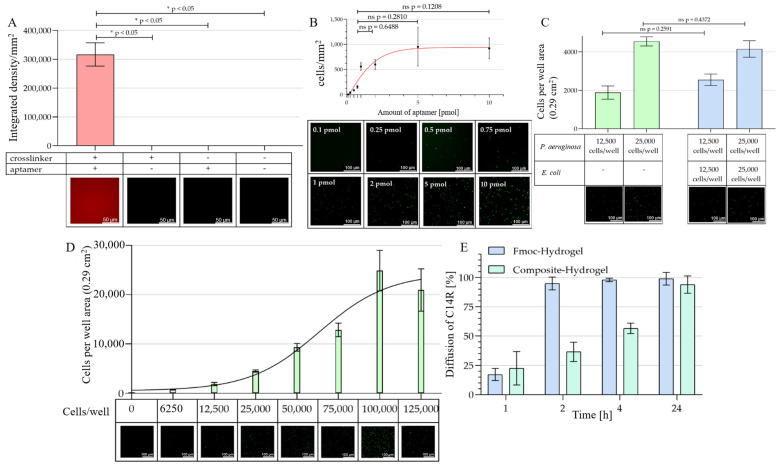
Evaluation of the AA-EraGel affinity functionality. (**A**) PEG_4_-SPDP crosslinker-mediated functionalization of BSA hydrogels with NH_2_-labeled anti-*P. aeruginosa* aptamers. To visualize the crosslinked aptamers on the BSA hydrogel, a Cy-5 labeled probe 5′[Cy5]-TCA AGT GGT CAT GTA CTA GTC AA-3′ was hybridized to the aptamers. Analysis of incomplete and fully functionalized BSA hydrogels was performed. Integrated density per area computed using FIJI software (version 1.53q) for each BSA hydrogel sample. The statistical analysis with a Mann–Whitney U-test shows significant *p*-values of *p* < 0.05 between the fully functionalized complex (+ +) and all other partially functionalized complexes. Fluorescence microscopy of complete functionalized BSA hydrogel, with crosslinker and aptamer (+ +), of BSA hydrogel with crosslinker but without aptamer (+ −), of BSA hydrogel without crosslinker but with added aptamer (− +), of only BSA hydrogel, without crosslinker and aptamer (− −) (magnification 400×). (**B**) Influence of the aptamer concentration used for BSA hydrogel functionalization on its binding capacity. Computed cells per mm^2^ for each BSA hydrogel condition for *P. aeruginosa* PAO1 pVLT-31 eGFP. The statistical analysis shows no significant *p*-values between the different amounts of aptamers used. Fluorescence microscopy of the fully functionalized BSA hydrogels with increasing amounts of aptamer and a surplus of GFP-labeled *P. aeruginosa* PAO1 pVLT-31 eGFP (10^6^ cells mL^−1^) (magnification 100×). (**C**) Specific binding of *P. aeruginosa* in the presence of *E. coli* as a “contaminating” control bacterium on aptamer-functionalized BSA hydrogels. A total of 12,500 and 25,000 cells per well were used. Computed cells per well area (0.29 cm^2^) for each BSA hydrogel for *P. aeruginosa* and *P. aeruginosa* + *E. coli* after half an hour of incubation. The statistical analysis shows no significant p-values between *P. aeruginosa* and the mix of *P. aeruginosa* + *E. coli*. Fluorescence microscopy of the functionalized BSA hydrogels with GFP-modified *P. aeruginosa* PAO1 pVLT-31 eGFP and *P. aeruginosa* PAO1 pVLT-31 eGFP + *E. coli* Nissle 1917 pVLT31-eGFP mix in comparison (magnification 100×). (**D**) Capacity analysis with the fully functionalized BSA hydrogels with different ratios of GFP-labeled *P. aeruginosa* cells. (**C**) Computed cells per well area (0.29 cm^2^) for each BSA hydrogel condition for *P. aeruginosa* PAO1 pVLT-31 eGFP after half an hour of incubation. (**D**) Fluorescence microscopy of the fully functionalized BSA hydrogels with increasing amounts of GFP-labeled *P. aeruginosa* PAO1 pVLT-31 eGFP (magnification 100×). (**E**) Cumulative release of AMP C14R from the drug zone and the drug zone plus carrier composite materials. The peptide-loaded materials were overlayed with PBS buffer and sequentially incubated for the given time periods. The cumulative concentrations of peptide measured as the absorption at 280 nm in the collected supernatants is given as the percentage of the peptide amount deposited in the respective material (1 h + 2 h + 4 h + 24 h). *P* values < 0.05 were considered significant. * denotes *p* < 0.05, ns: not significant.

We have recently introduced BSA crosslinked with EDC as a promising hydrogel material for biomedical applications, including functionalization with aptamers; however, this was in the form of hydrogel spheres in the range of approximately 1 mm [30,31]. Similar to our previous wound dressing publications, the intention here was to prepare larger-sized patches of composites to be subsequently not only loaded with drugs but also equipped with a specific affinity layer. Thus, we first demonstrated the successful functionalization of the BSA hydrogel layer with a focused library consisting of eight different individual aptamers against the surface structures of *P. aeruginosa* [29]. The synthesized aptamer library was used to functionalize the BSA hydrogel material with the help of the crosslinker PEG_4_-SPDP. In a two-step reaction, PEG_4_-SPDP first reacts with the thiol group of the BSA in a displacement reaction of the sulfur atom. In the second step, the NHS ester attacks the amino group of the modified aptamers. We tentatively started using 1.27 pmol per well, which reflects the calculated amount of BSA molecules being present on the surface of the hydrogel (theoretical 1:1 ratio). The successful binding of the aptamers to the surface was then verified by incubation with the complementary strand oligonucleotide fluorescently labeled with Cyanine-5 (Cy-5 reverse Primer), which can hybridize to the 3′- primer binding site of the aptamer molecules. Fluorescence labeling of the material was only achieved when both crosslinkers and aptamers were applied in the labeling reaction (Figure 2A). By contrast, when only one of the modifying agents was absent, the material was not stained, as was the case for the negative control represented by the pure material (Figure 2A).

To demonstrate the functionality of our concept, we then needed to show the binding of *Pseudomonas* cells to the material. We thus used a *P. aeruginosa* PAO1 pVLT-31 eGFP derivative, which expressed a recombinant GFP under transcriptional control of the tac-promoter of pVLT-31 as a fluorescence label upon induction with isopropyl-ß-D-thiogalactopyranosid (IPTG). The amount of aptamers was systematically increased from 0.1 to 10 pmol to functionalize the hydrogel surface in a well of a 96-well plate (0.29 cm^2^) to estimate the influence of the aptamer concentration on the resulting binding capacity. The observed obvious development of cell numbers bound along the increasing aptamer concentrations below 1 pmol did not, however, progress with a significant gain in the binding capacity at higher concentrations of aptamers used for functionalization. Interestingly, the theoretical 1:1 ratio of aptamer to BSA molecules on the material surface of a well (1.27 pmol) used in the initial experiment is perfectly close to the fitted non-linear regression of the results from the experiment shown in Figure 2B, suggesting that the 1:1 ratio does not generate a significant surplus of binding capacity compared to 1 pmol. We thus decided to use 1 pmol per well (3.45 pmol·cm^−2^ or 0.0345 pmol·mm^−2^) for the aptamer functionalization in the subsequent experiments.

Specificity testing revealed that the material perfectly bound *P. aeruginosa* cells in its completely functionalized form, whereas the GFP expressing the probiotic gut bacterium *Escherichia coli* Nissle 1917 pVLT31-eGFP, which served as a Gram-negative control bacterium, completely failed to bind to the material (Figure S1). This specificity was not limited when the cell number of the target bacterium was increased by 100%, even when the same high amount of the “contaminating” control bacteria was additionally present (Figure 2C).

The binding capacity of the aptamer-functionalized hydrogel was determined by depositing 200 μL of bacterial cell cultures adjusted to different and increasing numbers of cells in a fresh culture medium. The maximum of cells binding to the material was reached when 100,000 cells were applied and did not increase further with higher cell numbers. Under these conditions, i.e., with a liquid column of approximately 0.7 cm above the affinity material, 20,000 cells could be bound, which is 1/3 higher than the typical amount of bacteria in post-surgery wounds (calculated 14,500 cells per well area) (Figure 2D) [32].

After the characterization of the trapping zone of our intended composite material, we then had to demonstrate the functionality of the drug zone as a reservoir for antimicrobial peptides, their release from this reservoir and finally the complete functionality of the resulting composite material. First, the pure reservoir gel consisting of fibrillar Fmoc-protected phenylalanine was loaded with C14R to a final concentration of 40 μM and then covered with a 3x volume of assay buffer and incubated for 24 h. Samples were taken after 1, 2, 4 and 24 h and the peptide concentration in the assay buffer was determined to calculate the cumulative release of the peptide from the hydrogel reservoir. This gel showed the expected high release rate with the complete release already after two hours, whereas the composite showed a complete release after 24 h (Figure 2E) [33]. Fibrillary hydrogels of this type have been shown to be typically soft and have therefore been combined with mechanically supporting layers consisting of crosslinked BSA, which had the additional function of serving as attachment layers for binding molecules, equipping the resulting composite material with specificity toward selected pathogens [16,17]. Another important feature is that in combination this supporting layer does not only represent a not entirely impermeable barrier but allows an efficient diffusion to ensure a fast and even distribution of the drug molecules to be released. This is not only a relevant property with respect to later industrial processes of such materials but a homogeneously drug-preloaded affinity layer also results in instant pharmacological effects for a convenient topical application of the final wound dressing material.

**Figure 3:**
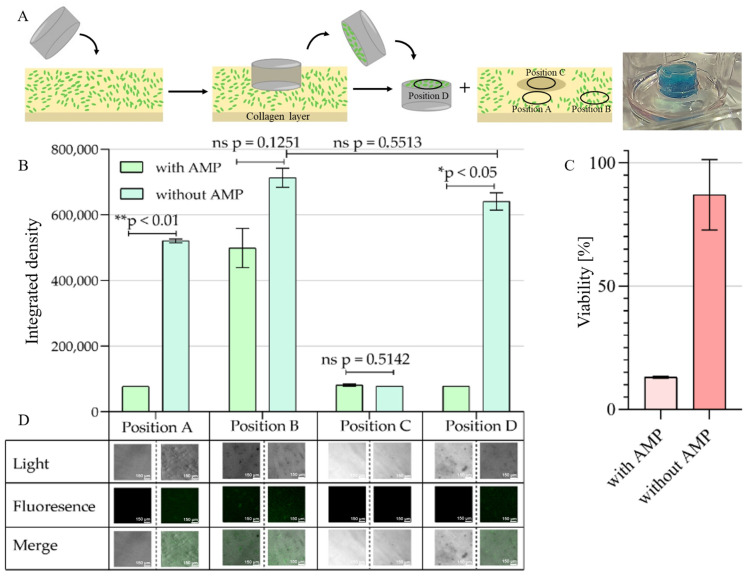
AA-EraGel-mediated removal and eradication of *P. aeruginosa* on a collagen matrix: proof-of-concept. (**A**) A collagen-based hydrogel mimicking a naturally occurring extracellular matrix for binding of pathogenic cells was “infected” with *P. aeruginosa* cells. The composite hydrogel loaded with C14R (10 μg × mL^−1^) was applied directly onto the *P. aeruginosa* cells with the trapping surface facing toward the collagen. (**B**) Computed integrated density per area of the fluorescent microscopy by FIJI software (version 1.53q) for each position. Position A: in close proximity to the composite hydrogel. Position B: far remote from the application site. Position C: directly under the application site. Position D: Trapping surface of the wound dressing material. Statistical analysis was performed with a Mann–Whitney U-test. (**C**) Viability of *P. aeruginosa* was visualized using a resazurin live/dead assay of the trapped cells on Position D. (**D**) Fluorescence microscopy and phase-contrast microscopy for each position at 630× magnification. The photograph (top right) shows the experimental situation with the wound dressing composite lying in contact with the collagen matrix. To increase visibility, the top layer in direct contact with the collagen surface has been stained in blue with Indigotin I (E 132) (Erich Wutzig Inh. Andreas Saf, Sitzendorf, Germany). *p* values < 0.05 were considered significant. * denotes *p* < 0.05, ** <0.01, ns: not significant.

The ultimate goal of the AA-EraGel affinity trap concept is the application of the hydrogel-based composite for wound care applications for high-risk patients or in high-risk hospital environments such as intensive care units, in which immediate post-surgery infections with severe microbial pathogens must be prohibited to protect patients from the establishment of persisting and life-threatening infections. This involves the removal of *P. aeruginosa* cells from the wound surface immediately after the onset of infection during or after the completion of surgery and, as the second key step, the subsequent killing of the pathogen safeguarding the total eradication of the bacteria. This dual mode of action represents a combined first defense line unifying simple binding and subsequent mechanical removal from the wound surface and early pharmaceutical inhibition of the establishment of an infection by cells remaining on the wound surface. As a first and simple experimental step to approaching this goal, we decided to use a collagen hydrogel as a model surface mimicking a naturally occurring extracellular matrix for the binding of pathogenic cells to which the affinity trap composite could be directly applied afterward. In this idealized early post-infection wound care situation, four regions (Figure 3A) of the large-area wound and the contact areas between the wound surface and wound dressing are of relevance for analyzing the success of pathogen removal and killing. Whereas in close proximity to the application site (Position A) an antimicrobial effect of the AMP can be expected due to its diffusion, this declines with the distance to the application site resulting in an undisturbed development of the pathogen far remote from the application site (Position B). Most important in this context is the area of the wound being in direct contact with a wound dressing, consisting of the respective wound surface (Position C) and the opposite surface of the wound dressing material itself, which after removal will carry attached cells in the case of successful high-affinity binding to the material (Position D). To reflect this situation, a simulated extracellular wound matrix was created on the bottom of a 12-well microtiter plate by coating it with collagen from rat tails, which was then “infected” with an excess of *P. aeruginosa* cells in our experimental setup. To simulate an immediate post-surgery infection, cells were forced toward the collagen matrix by gentle centrifugation and allowed to establish physical contact for 30 min. The AA-EraGel model wound-dressing patch consisting of the peptide-loaded affinity composite hydrogel trap, or the empty version serving as a negative control focusing only on the affinity aspect, were then applied to the “infected” wound surface for 24 h to exhibit their respective impacts on the pathogenic cells. The wound dressing patches were then removed and the number of cells present on the four relevant regions after this procedure was quantified by measuring the integrated density of fluorescence and, in the case of positions B and D (i.e., far away from the contact area as a control and directly on the surface of the wound-dressing patch, respectively), by the resazurin-based viability assay. Meeting the expectation, the killing effect of C14R on *P. aeruginosa* cells residing at positions A and B was more pronounced for the loaded material close to the patch but reduced at a larger distance, where the difference between the peptide-loaded material and the empty control turned out to be not significant (Figure 3B). Directly under the wound dressing (Position C), no distinct cells were visible in fluorescence microscopy independent from the material variant used. This is an indication that contact with the material surface without applying the antimicrobial peptide is sufficient to quantitatively remove bacterial cells from the wound surface model. Interestingly, both position C regions showed residual fluorescence, which may be caused by cell lyses upon contact with the wound dressing and appears to be independent of the presence of the peptide (Figure 3B). By contrast, on the former wound-oriented surface of the removed patch (Position D) cells were present in numbers similar to the unaffected regions B, which were significantly reduced in the presence of the peptide (Figure 3B). The fluorescence microscopy analysis was then complemented by the resazurin-based live/dead assay on the residual cells of Position D, which confirmed that the peptide effectively killed *P. aeruginosa* on the wound dressing surface (Figure 3C).

## 3. Discussion

“Smart wound dressings” as a concept to treat severe and predominantly chronic wounds (e.g., in the context of diabetes) in the future were introduced in the early 2000s, with a logarithmic rise in publication numbers leading to several hundred per year at present [34]. By definition, the development of such materials or devices involves different scientific disciplines including engineering, chemistry, biology, physics, or medical science, explaining why the main focus on what is interesting and “smart” can be extremely diverse. Materials have been developed which allow the monitoring of the wound status to evaluate wound healing and the presence or onset of potentially harmful infections, through different technologies including fluorescence-based techniques and the implementation of electronic components for signal generation and transduction to monitoring devices [35,36,37,38]. Another main aspect is to promote wound healing or protect wounds from being negatively affected by infections with pathogenic microbes. Wound dressing materials have been invented which can release growth factors or antimicrobial drugs including antimicrobial peptides [16,17,39,40]. We have recently enlarged the portfolio of options by introducing the so far underrepresented aspect of manipulating early phases of wound infections by providing affinity functions against pathogens to the wound dressing material by decorating the surface of hydrogel composites with microbial multivalent lectins [16,17,24,41,42]. However, the lectin-mediated binding is not limited to pathogenic yeasts or bacteria but can also immobilize human cells in vitro and exert potentially considerable adverse biological effects on wound healing in vivo [43,44]. In contrast to relatively unspecific lectins, aptamers as a versatile class of binding molecules are gaining a remarkable degree of scientific prominence for their broad spectrum of dedicated targets, which is not limited to small molecules or proteins but can include with high specificity and robustness the binding and recognition of whole cells, tissues and organs [45,46,47,48]. The group of whole cells as potential aptamer targets consists of both pathogenic or probiotic Gram-positive and Gram-negative bacteria, as well as other prominent pathogens such as yeasts from the *Candida* genus [29,49,50,51,52,53,54,55]. The aptamer technology has been used to develop various aptamer-based biosensors for clinical diagnostics, food, and environmental monitoring of *P. aeruginosa* in recent years (nicely reviewed in Zheng et al., 2020) [56]. We have previously shown that in contrast to individually selected aptamer molecules, it may be advantageous to use polyclonal aptamer libraries for robust and secure binding of *P. aeruginosa* cells [29]. These libraries were further developed into a focused library consisting of eight different aptamers and allowed specific and reliable binding of the pathogen on hydrogel-based surfaces in serum and blood [30]. Our previous work showed that composite hydrogels consisting of BSA-derived materials and fibrillar hydrogels composed of the Fmoc-protected amino acids phenylalanine and methionine were suited to developing possible next-generation wound dressing materials with respect to their mechanical, rheological and diffusion properties [16,17,25,33]. The functionality of such materials was demonstrated by efficiently delivering the antimicrobial peptides Cm-p5 and C14R to pathogenic *Candida* spec. or *P. aeruginosa* cells residing on their surfaces [16,17,23,57]. To our knowledge, so far, aptamers have not been implemented as affinity-mediating molecules on biomaterials intended for applications in wound care. We thus wanted to show that the specificity of the focused anti-*P. aeruginosa* library can be used to provide capturing functionality on the AA-EraGel basic wound care hydrogel composite. Functionalization of the BSA-based trapping zone top layer resulted in specific and efficient immobilization of the target cells. This was also the case for cells residing on the surface of a collagen-based gel mimicking, as an extremely simplified model, the matrix of a fresh wound. In this model, *P. aeruginosa* cell numbers could be removed with a capacity higher than the typical count of bacteria in post-surgery wounds [32]. The viability assays performed with the cells sticking to the removed model AA-EraGel wound dressing patch showed that as expected the residual cells were killed by the C14R peptide. Thus, we reasoned that the application of AA-EraGel to the model wound allows not only the sufficient quantitative removal of *P. aeruginosa* from freshly infected surfaces in a significant amount but can also be strengthened by delivering antimicrobial drugs to the trapped pathogens. In this respect, the drug delivery represents a safeguarding measure, which may have additional importance, especially if intermediate or long-term dwell times of the final wound care materials in hospital environments or at patient bedsides at home are intended. We believe that the use of highly specific binding molecules such as the anti-*P. aeruginosa* aptamers used in the first example presented here may open new avenues toward next-generation wound dressing materials with respect to specificity and efficiency to control or prevent the establishment of harmful (hospital-acquired) infections by removing the infective agent from the treated wound. We also believe that functionalization with aptamers is not only not limited to our model hydrogel materials but can also be attractive in combination with novel biomaterials and innovative wound care applications. The implementation of aptamer-based optical or electronic sensing platforms into wound care materials, or the analysis of binding events of pathogens to affinity materials with such sensors alone, represent logical and attractive further opportunities [58,59,60]. Examples of interest on the alternative material side may be polydopamine films, which have been successfully introduced as wound dressing constituents (nicely reviewed in Alfieri et al., 2022 and Yazdi et al., 2022) [61,62]. Pathogen-specific aptamers may be further developed as attractive molecular binding entities and important affinity mediators in the toolbox of materials, techniques and drugs which in integrated novel material design approaches can help to deliver new gentle and efficient wound care and skin regeneration options in healthcare environments.

## 4. Materials and Methods

### 4.1. Specific Anti-P. aeruginosa PAO1 Aptamer PCR

The eight specific aptamers against *P. aeruginosa* PAO1 (C1R1, C2R1, C2R2, C2R10, C4R2, C6R3, C10R5 and C10R6), which were already characterized in Kubiczek et al., 2020, were produced by PCR. An NH_2_-labeled primer ((5′[NH2]-TAG GGA AGA GAA GGA CAT ATG AT-3′), Eurofins Genomics Germany GmbH) and a biotin-labeled reverse primer ((5′[Biotin]-TCA AGT GGT CAT GTA CTA GTC AA-3′), Eurofins Genomics Germany GmbH, Ebersberg, Germany) were used [29]. The following was utilized for each PCR: 1x PCR buffer (1.5 mM MgCl_2_), 250 µM of each dNTP, 0.25 µM of the labeled primers and 0.2 µM aptamer ssDNA, Herculase II Fusion DNA Polymerase (Agilent Technologies, Inc., Santa Clara, CA, USA). Amplification was performed in the thermocycler SensoQuest Labcycler (SensoQuest GmbH, Göttingen, Germany) with an initial denaturation step at 94 °C for 3 min, followed by 25 cycles at 94 °C for 30 s for denaturation, 49.1 °C for 30 s for annealing, 72 °C for 30 s for elongation and a final extension at 72 °C for 2 min. Subsequently, the respective PCR products for each aptamer were pooled and gel electrophoresis (gel with 2% agarose in 0.5% TBE buffer) was performed. After loading the gel with 5 µL of dsDNA from each sample and 1 µL of 6x TriTrack DNA Loading Dye (Thermo Fisher Scientific, Inc., Waltham, MA, USA), it was performed at 150 V for 35 min and then stained in a 0.007% ethidium bromide bath and viewed under UV light (E-Gel^®^ Imager, Thermo Fisher Scientific, Inc., Waltham, MA, USA).

### 4.2. Preparation of Aptamer ssDNA

The preparation of the ssDNA aptamers was performed as in Krämer et al., 2021, already described to be further used on the hydrogel construct [30]. Here, 50 µL of streptavidin-coated magnetic beads were washed three times with 1 mL of 1× DPBS using a magnetic separator. Amplified aptamer dsDNA was then added, covered and incubated at 22 °C and 50 rpm for 16 h. To remove unbound dsDNA, the supernatant was removed, and the magnetic streptavidin beads were washed with 1 mL of 1× DPBS. After that, 50 µL of NaOH (100 mM) was added to the magnetic streptavidin beads and incubated for 2 min without magnetic separation. Then, 45 µL of the NaOH solution was transferred to a new reaction tube containing 126 µL of 1× DPBS and 34.4 µL of NaH_2_PO_4_ buffer (100 mM). The remaining 5 µL of ssDNA was used for separation by gel electrophoresis. After gel electrophoresis, the ssDNA concentration was measured in ng µL^−1^ using an Implen NP80 nanophotometer (Implen GmBH, Munich, Germany).

### 4.3. Synthesis of AMP

A derivative of BP100, named C14R, with the amino acid sequence CSSGSLWRLIRRFLRR was synthesized on a 0.10 mmol scale via standard Fmoc solid phase peptide synthesis techniques. This was achieved with a preloaded serine resin and washed with dimethylformamide (DMF). To remove the protecting group of Fmoc, 20% (*v*/*v*) piperidine in DMF which was initialized with microwaves was used. After that, an additional washing step with DMF was performed. Subsequently, the addition of amino acids in 0.2 mol equiv. to the reactor was carried out, followed by HBTU 2-(1H-benzotriazol-1-yl)-1,1,3,3-tetramethyluronium-hexafluorophosphate) in a 0 and 5 mol equiv. into the amino acid solution. Then, 2 mol equiv. of N,Ndiisopropylethylamine (DIEA) was added. The coupling reaction was achieved via microwaves within minutes. The resin was again washed in DMF. This procedure was repeated for all amino acids in the sequence. After completion of the amino acid synthesis, the peptide was cleaved in 95% (*v*/*v*) trifluoracetic acid (TFA), 2.5% (*v*/*v*) triisopropylsilane (TIS) and 2.5% (*v*/*v*) H2O for 1 h. A precipitation of the peptide residue and washing with cold diethyl ether (DEE) by centrifugation was carried out which was then under vacuum to remove the residual ether. To purify the peptide, reversed phase preparative high-performance liquid chromatography (Waters GmbH, Eschborn, Germany) was used in an acetonitrile/water gradient under acidic conditions on a Phenomenex C18 Luna column (5 mm pore size, 100 Å particle size, 250–21.2 mm). Liquid chromatography–mass spectroscopy (Waters GmbH, Eschborn, Germany) was used to measure the peptide mass.

### 4.4. Bacteria Cultivation

Two strains were used in the experiments: *P. aeruginosa* PAO1 pVLT31-eGFP and *E. coli* Nissle 1917 pVLT31-eGFP. For the cultivation of each bacterial strain, precultures were prepared in 5 mL of LB medium (Carl Roth GmbH + Co. KG, Karlsruhe, Germany) containing 10 µg mL^−1^ of tetracycline (Carl Roth GmbH + Co. KG, Karlsruhe, Germany). These precultures were incubated at 37 °C for 16 h while shaking at 150 rpm. The next day, the OD_600_ values of the precultures were determined, and a flask containing 25 mL LB medium and 10 µg mL^−1^ tetracycline was subsequently inoculated with an initial OD_600_ of 0.05. Cultivation of the flasks was performed at 37 °C with shaking (150 rpm). Upon reaching an OD_600_ of 0.6, cultures were induced with 0.4 mM isopropyl-ß-d-1-thiogalactopyranoside (IPTG, Carl Roth GmbH + Co. KG, Karlsruhe, Germany). The bacterial cultures were then incubated for up to three more hours until the stationary phase was reached. In order to achieve the desired cell numbers per mL in the subsequent experiments, the cells were counted under the microscope (Leica Microsystems CMS GmbH, Wetzlar, Germany) using a Thoma cell counting chamber (LO-Laboroptik GmbH, Friedrichsdorf, Germany).

### 4.5. BSA Hydrogel and Functionalization with Aptamer ssDNA

The BSA hydrogels were prepared as reported previously; in brief, two stock solutions were prepared, a 20% (*w*/*v*) BSA- (neoFroxx GmbH, Einhausen, Germany) and a 10% (*w*/*v*) EDC-solution (Carl Roth GmbH + Co. KG, Karlsruhe, Germany), both diluted in MES (2-(*N*-morpholino)ethanesulfonic acid) buffer (100 mM) [16,17,30]. These were then mixed in a one-to-one ratio (40 µL) in a 96-well plate. In the next step, PEG_4_ SPDP linker (4-unit polyethylene glycol spacer arm, with an amine-reactive N-hydroxysuccinimide (NHS) ester at one side and a sulfhydryl-reactive 2-pyridyldithiol group at the other end) (Thermo Fisher Scientific, Waltham, MA, USA) (6.5 μL, 0.2 μM in DMSO) was added and filled up with 200 µL PBS-EDTA and incubated for 16 h at room temperature, followed by extensive washing twice with 200 µL of PBS-EDTA. The eight aptamers were prepared in the desired concentrations and activated to ensure the correct folding in PBS-EDTA. They were heated to 95 °C for 5 min, cooled for 5 min on ice, and then the aptamer solution was covered and stored at RT for 30 min to facilitate the correct folding of the aptamers. The native 3D structure of the aptamers can be ensured after complete denaturation and direct refolding. The aptamer solution was covered and stored at RT for 30 min. After that, the aptamer solution was added to the well and filled up to 200 µL with PBS-EDTA buffer, and then incubated while covered for 1 h at RT. After incubation, the aptamer-crosslinked BSA hydrogels were washed 3 times with 200 µL PBS-EDTA buffer.

### 4.6. Peptide Hydrogel and AMP Loading

To prepare the peptide hydrogel, Fmoc-phe-OH was diluted in DMSO (100 mg mL^−1^) and then mixed with phosphate buffer (10 mM Na_2_HPO_4_; 42 mM NaH_2_PO_4_ in demin water, pH 7.4) in a ratio of 12.5:1, as described previously [17]. They were also prepared in a 96-well plate (50 µL). To load the hydrogels with AMP, the appropriate volume of AMP was added to the phosphate buffer to obtain a concentration of 10 μg mL^−1^. The hydrogels were stored at 4 °C overnight.

### 4.7. Composite Hydrogel

BSA and peptide hydrogels were prepared as described above. The peptide hydrogel, which was loaded with AMP, was cast first and polymerized overnight at 4 °C. The BSA hydrogel, which polymerizes very fast, was placed on top of the peptide hydrogel the next day and functionalized with aptamers.

### 4.8. Verification of the Functionalized BSA Hydrogels

To visualize the crosslinked aptamers on the BSA hydrogel, a Cy-5 labeled probe (5′[Cy5]-TCA AGT GGT CAT GTA CTA GTC AA-3′] was hybridized to the aptamers crosslinked to the hydrogels. Various approaches have been taken for this purpose. The complete construct (functionalized with crosslinker PEG_4_-SPDP and aptamer as described in Section 4.5) was used as well as hydrogels without aptamer or without crosslinker, as well as without both. Subsequently, after the preparation of the various constructs, the probe was activated as described for the aptamers in 4.5. Then, 1.27 µL of the 1 pmol µL^−1^ activated Cy-5 labeled probe was pipetted onto the gels. For hybridization, the hydrogel was covered with 200 µL PBS-EDTA buffer and incubated under light exclusion overnight at 4 °C. The next day, unattached probe remnants were removed by washing three times with 200 µL PBS-EDTA buffer. Afterward, the hydrogels were examined by fluorescent microscopy at 400× magnification with the Leica DMi8 inverted fluorescent microscope (Leica Microsystems CMS GmbH, Wetzlar, Germany). Images of the different conditions were taken and analyzed with FIJI image analysis software [63].

### 4.9. Binding Specificity Analysis of P. aeruginosa PAO1 pVLT31-eGFP with Different Aptamer Concentrations

The influence of the aptamer concentration used for BSA hydrogel functionalization on its binding capacity was investigated in the following experiment. BSA hydrogels were prepared as described in Section 4.5. After the first functionalization with the crosslinker PEG_4_-SPDP and washing 3 times with 200 µL PBS-EDTA, the desired amounts of 0.1, 0.25, 0.5, 0.75, 1, 2, 5 and 10 pmol of NH_2_-labeled aptamer were added to the gels for complete functionalization. Then, the samples were incubated while covered for 1 h at RT. After incubation, the BSA hydrogels were washed 3 times with 200 µL PBS-EDTA. In the next step, *P. aeruginosa* cells/200 µL (106 cells mL^−1^) were pipetted to each sample and incubated for 30 min while covered at RT. Then, the gels were washed 3 times with 200 µL PBS-EDTA and filled up to 200 µL with PBS-EDTA. In the next step, all samples were investigated with fluorescence microscopy at 100× magnification. After that, the microscopy images were analyzed with FIJI [63].

### 4.10. Binding Specificity Analysis of P. aeruginosa PAO1 pVLT31-eGFP to Incomplete and Fully Functionalized BSA Hydrogel

To determine the specific binding of *P. aeruginosa* PAO1 pVLT31-eGFP against the crosslinked aptamers to the BSA hydrogels, various constructs as described in Section 4.8 were used. Therefore, BSA hydrogels from Section 4.5, and *P. aeruginosa* PAO1 pVLT31-eGFP bacteria and *E. coli* Nissle 1917 pVLT31-eGFP bacteria, as a control strain, from Section 4.4, were used. The first experiment had the fully functionalized hydrogel construct. The second control experiment had an incomplete functionalization construct without NH_2_ aptamers. The third construct was crosslinked for 16 h with PEG_4_-SPDP but was not functionalized with NH_2_-labeled aptamers, and the last construct was without both crosslinker and aptamer. After washing 3 times with 200 µL PBS-EDTA, 25,000 bacterial cells/200 µL of *P. aeruginosa* PAO1 pVLT31-eGFP or *E. coli* Nissle 1917 pVLT31-eGFP were pipetted into the wells and incubated while covered for 30 min at RT. Afterward, all constructs were washed 3 times with 200 µL PBS-EDTA and then filled up to 200 µL with PBS-EDTA. The samples were then examined by fluorescence microscopy at 100× magnification with the Leica DMi8 inverted fluorescent microscope. Fluorescence microscopy images were analyzed with FIJI [63].

### 4.11. Binding Specificity Analysis of P. aeruginosa PAO1 pVLT31-eGFP in Presence of a “Contaminating” Control Bacteria

To show that other bacteria have no influence on the binding of *P. aeruginosa* to the hydrogel trap, *E. coli* Nissle 1917 pVLT31-eGFP bacteria were used to produce bacterial mixtures with *P. aeruginosa* PAO1 pVLT31-eGFP. For this purpose, the hydrogels were first functionalized as in Section 4.5. After functionalization, precultures of *P. aeruginosa* and *E. coli* were prepared as in Section 4.4, the bacteria cells were counted, and different ratios of 12,500 cells/200 µL and 25,000 cells/200 µL per bacterium were used. In the next step, the samples were covered and incubated for 30 min at RT, washed 3 times with 200 µL PBS-EDTA and then filled up to 200 µL with PBS-EDTA. All samples were examined with fluorescence microscopy at 100× magnification, and images were analyzed with FIJI [63].

### 4.12. Binding Capacity of P. aeruginosa PAO1 pVLT31-eGFP

For the subsequent experiments, BSA hydrogels were prepared as described in Section 4.5 in a 96-well plate. After full functionalization with PEG_4_- SPDP and 1 pmol NH_2_-labeled aptamer, the gels were washed 3 times with 200 µL PBS-EDTA. In the following step, 6,250, 12,500, 25,000, 50,000, 75,000, 100,000 and 125,000 cells/200 µL of *P. aeruginosa* PAO1 pVLT31-eGFP were pipetted to the fully aptamer-functionalized hydrogels. After covered incubation for 30 min at RT, the hydrogels were washed 3 times with 200 µL PBS-EDTA and then filled up to 200 µL with PBS-EDTA. In the next step, all samples were examined with fluorescence microscopy at 100× magnification, and all images were analyzed by FIJI [63].

### 4.13. Protein Release of AMP C14R

For the subsequent experiments, AMP-loaded hydrogels were prepared as described in Section 4.6 in a 96-well plate. AMP-loaded hydrogels were covered with 200 µL PBS. Samples were taken at regular intervals (1 h, 2 h, 4 h and 24 h) and then recovered with 200 µL PBS. The absorption of the samples was measured at 280 nm with a Tecan Spark microplate reader (Tecan Group Ltd., Männedorf, Switzerland). All measurements were performed in triplicate. The error bars represent the standard deviation.

### 4.14. Collagen Matrix Model

A collagen model was prepared for wound care. For this, 150 µL of a stock solution of 1 mg mL^−1^ collagen from rat tail in acetic acid and 10x PBS with 1.5 μL NaOH (1M) was poured into a 24-well plate. Gelation was performed for 1 h at 30 °C. Subsequently, 10^6^ cell mL^−1^ of *P. aeruginosa* bacteria were centrifuged onto the collagen matrix and incubated while covered for 30 min at RT. The composite hydrogel loaded with C14R (10 μg mL^−1^) was placed on the collagen layer by removing it from the mold with tweezers, facing down with the affinity layer to the cells. After 24 h of incubation, the composite hydrogel was taken off and the affinity layer as well as the collagen matrix, in close proximity as well as far away and under the wound dressing, were examined with fluorescence microscopy at 10× magnification, and all images were analyzed with FIJI [63].

### 4.15. Viability Test: Resazurin Assay

A resazurin assay was performed on the wound patch to test cell viability. The composite hydrogel was covered after incubation on the collagen matrix with 200 μL resazurin (0.15 mg mL^−1^). After two hours of incubation, the amount of metabolized fluorescent resorufin was measured with a Tecan200 M fluorescence reader (Tecan Group Ltd., Mannedorf, Switzerland) at an excitation wavelength of 535 nm and an emission wavelength of 595 nm.

### 4.16. Statistical Analysis

Statistical analysis was performed with a Mann–Whitney U-test [64].

## Data Availability

Not applicable.

## Supplementary Materials

The following supporting information can be downloaded at: https://www.mdpi.com/article/10.3390/ijms24054800/s1.
