# Supplementary material for: Aptamers as Novel Binding Molecules on an Antimicrobial Peptide-Armored Composite Hydrogel Wound Dressing for Specific Removal and Efficient Eradication of Pseudomonas aeruginosa
Source: Int J Mol Sci. 2023 Mar 2;24(5):4800. doi: 10.3390/ijms24054800 (PMC10002764; doi:10.3390/ijms24054800)
Supplement: Supplementary file 1 [file ijms-24-04800-s001.zip › ijms-2208123-supplementary.pdf]

International Journal of *Molecular Sciences*

MDPI

Communication

Aptamers as Novel Binding Molecules on an Antimicrobial Peptide-Armored Composite Hydrogel Wound Dressing for Specific Removal and Efficient Eradication of Pseudomonas aeruginosa

Markus Kraemer [1,†], Magali Bellion [1,†], Ann-Kathrin Kissmann [1,2,*], Tilmann Herberger [2], Christopher V. Synatschke [2] , Anil Bozdogan [3,4], Jakob Andersson [4], Armando Rodriguez [5,6] , Ludger Ständker [6] , SebastienWiese [6], Steffen Stenger [7], Barbara Spellerberg [7], Kay-Eberhard Gottschalk [8], Ahmet Cetinkaya [9] , Joanna Pietrasik [9] , Tanja Weil [2] and Frank Rosenau [1,2,*]

1 Institute of Pharmaceutical Biotechnology, Ulm University, Albert-Einstein-Allee 11, 89081 Ulm, Germany

2 Max-Planck-Institute for Polymer Research Mainz, Ackermannweg 10, 55128 Mainz, Germany

3 Center for Electrochemical Surface Technology (CEST), Austrian Institute of Technology, 3420 Tulln, Austria

4 Austrian Institute of Technology, Giefinggasse 4, 1210 Vienna, Austria

5 Core Facility for Functional Peptidomics, Ulm Peptide Pharmaceuticals (U-PEP), Faculty of Medicine, Ulm University, 89081 Ulm, Germany

6 Core Unit of Mass Spectrometry and Proteomics, Faculty of Medicine, Ulm University, 89081 Ulm, Germany

7 Institute for Medical Microbiology and Hygiene, University Hospital Ulm, 89081 Ulm, Germany

8 Institute of Experimental Physics, Ulm University, Albert-Einstein-Allee 11, 89081 Ulm, Germany

9 Institute of Polymer and Dye Technology, Lodz University of Technology, Stefanowskiego 16, 90-537 Lodz, Poland

* Correspondence: ann-kathrin.kissmann@uni-ulm.de (A.-K.K.); frank.rosenau@uni-ulm.de (F.R.)

† These authors contributed equally to this work.

Supplementary Materials

A

B

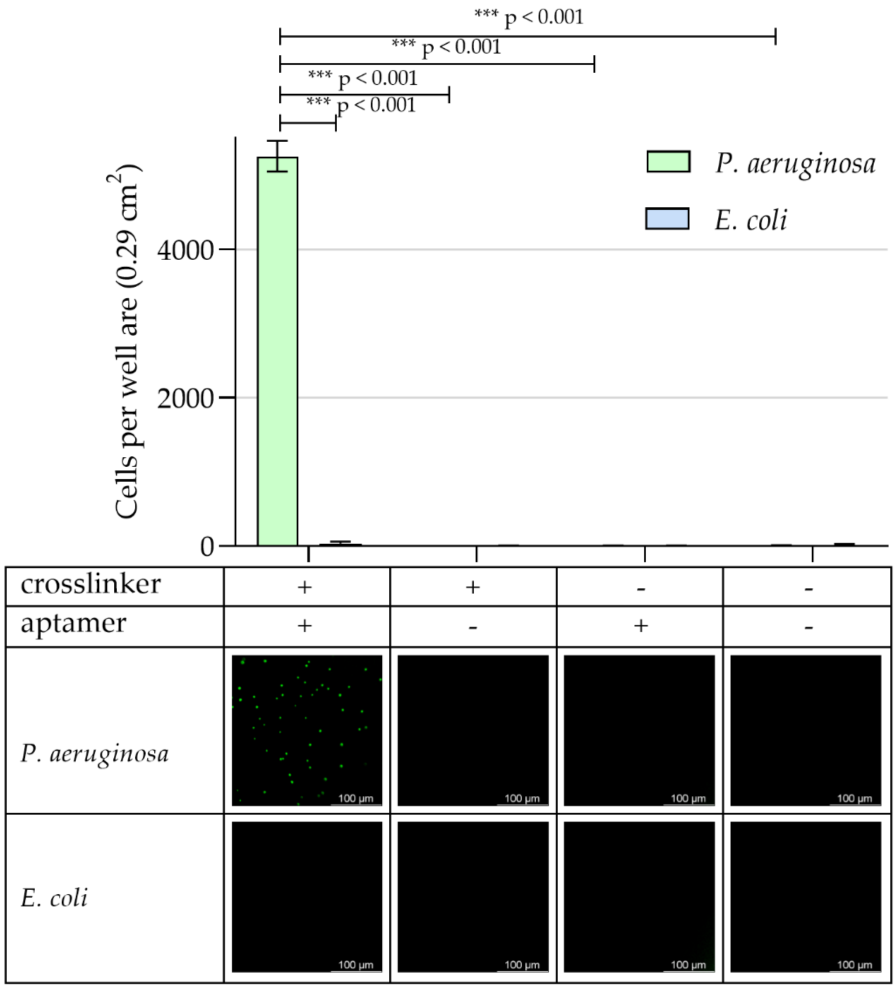

Figure S1. Analysis of the specific binding of *P. aeruginosa* PAO1 and *E. coli* to incomplete and fully functionalized BSA hydrogels. 25,000 cells were used on the complete and incomplete BSA hydrogels. (A) Computed cells per mm² for each BSA hydrogel condition for *P. aeruginosa* PAO1 and *E. coli.* The statistical analysis with a Mann-Whitney-U-Test shows significant p-values of $p < 0.001$ between the fully functionalized complexes with *P. aeruginosa* and *E. coli* and all other partially functionalized complexes with *P. aeruginosa*. + denotes presence and – denotes absence of components. (B) Fluorescence microscopy of the functionalized BSA hydrogels with GFP-modified *P. aeruginosa* and *E. coli* cells in comparison to incomplete functionalized BSA hydrogels (magnification 100x). *P* values < 0.05 were considered significant. *** denotes $P < 0.001$.
